# Supplementary material for: Shelf-Life Prediction and Critical Value of Quality Index of Sichuan Sauerkraut Based on Kinetic Model and Principal Component Analysis
Source: Foods. 2022 Jun 15;11(12):1762. doi: 10.3390/foods11121762 (PMC9222660; doi:10.3390/foods11121762)
Supplement: Supplementary file 1 [file foods-11-01762-s001.zip › foods-1723899-SI.pdf]

Article

Shelf life prediction and critical value of quality index of Sichuan sauerkraut based on kinetic model and principal component analysis

Jie Du[a,b], Min Zhang[a,c*], Lihui Zhang[a], Chung Lim Law [d] and Kun Liu[e]

[a] State Key Laboratory of Food Science and Technology, School of Food Science and Technology, Jiangnan University, 214122Wuxi, Jiangsu, China
[b] International Joint Laboratory on Food Safety, Jiangnan University, 214122 Wuxi, Jiangsu, China
[c] Jiangsu Province Key Laboratory of Advanced Food Manufacturing Equipment and Technology, Jiangnan University, China
[d] Department of Chemical and Environmental Engineering, University of Nottingham, Malaysia Campus 43500 Semenyih, Selangor, Malaysia
[e] Sichuan Tianwei Food Group Co., Ltd., 610000 Chengdu, China
*Correspondence: author: State Key Laboratory of Food Science and Technology, Jiangnan University, Wuxi, China. Tel./fax: +86 510 85877225 (M. Zhang). Correspondence: min@jiangnan.edu.cn (M. Zhang)

Abstract: Sichuan sauerkraut is a kind of fermented vegetable, and a series of physicochemical changes occurred in Sichuan sauerkraut during storage. Kinetic models were used to describe the changes in the physicochemical properties of Sichuan sauerkraut and predict shelf life. The texture, color, total acid, microbe, near infrared, volatile components, taste and sensory evaluation of Sichuan sauerkraut during storage at 25℃, 35℃ and 45℃ were studied. Principal component analysis (PCA) and Fisher discriminant analysis (FDA) were used to analyze the e-tongue data. According to the above analysis, Sichuan sauerkraut with different storage time can be divided into three types：completely acceptable period, acceptable period and unacceptable period. Through this classification method, the critical values of various quality indicators can be determined more accurately. It was found that the zero-order kinetic reaction model (R^2, 0.8699-0.9895) has a higher fitting degree than the first-order kinetic reaction model. The Arrhenius model (E_a value is 47.23-72.09 kJ/mol, k_{ref} value is 1.076×10^6-9.220×10^{10} d^{-1} has higher fitting degree than Eyring model. The combination of zero-order kinetic reaction model and Arrhenius model could better predict the physical indexes of Sichuan sauerkraut, and the error back propagation artificial neural network (BP-ANN) model could better predict the chemical indexes. It is a better choice for dealers and consumers to judge the shelf life and edibility of food by shelf life model.

Keywords: Reaction kinetics model; Arrhenius model; BP-ANN; total acid; E-tongue; GC-MS

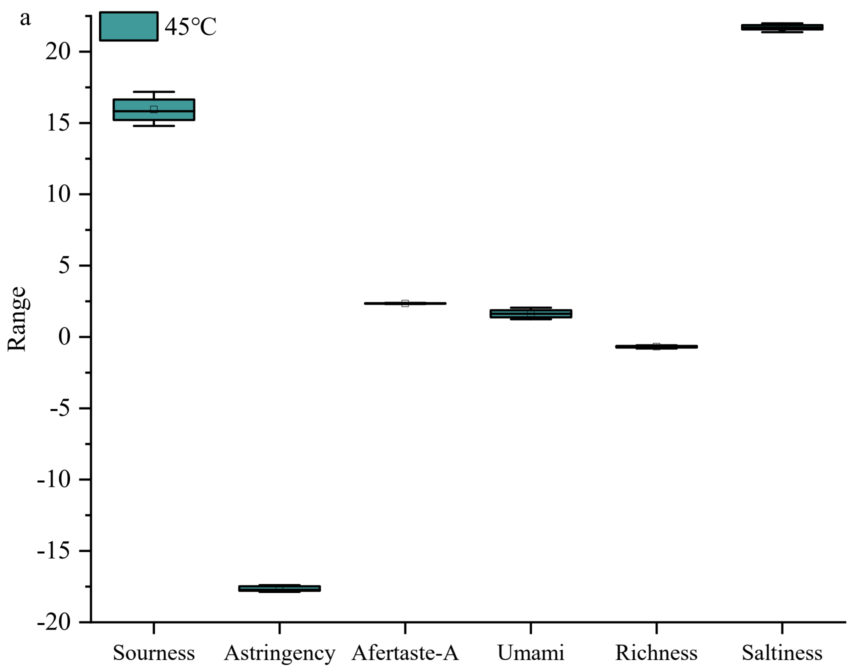
a
45℃
Range
20
15
10
5
0
-5
-10
-15
-20
Sourness
Astringency
Afertaste-A
Umami
Richness
Saltiness

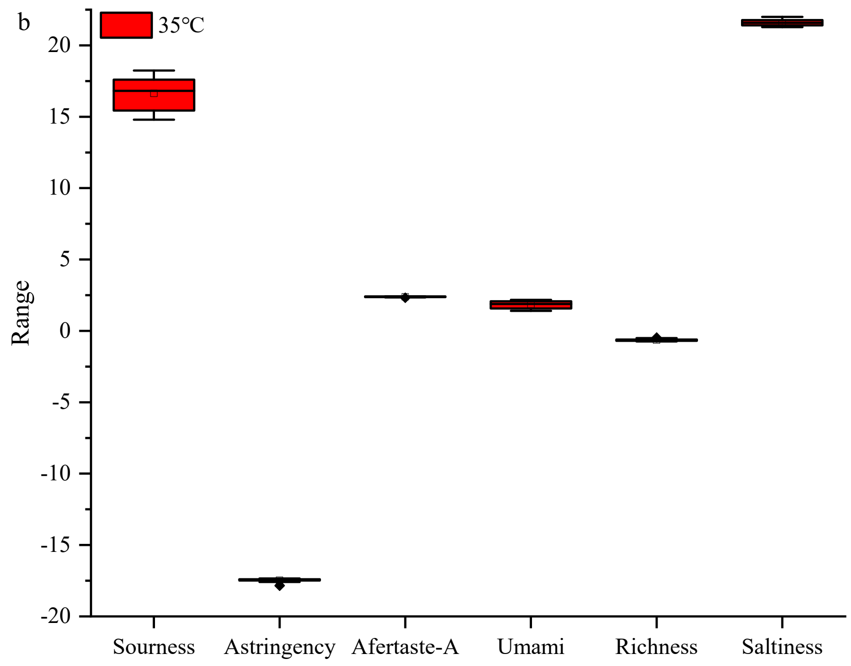
b
35℃
Range
20
15
10
5
0
-5
-10
-15
-20
Sourness
Astringency
Afertaste-A
Umami
Richness
Saltiness

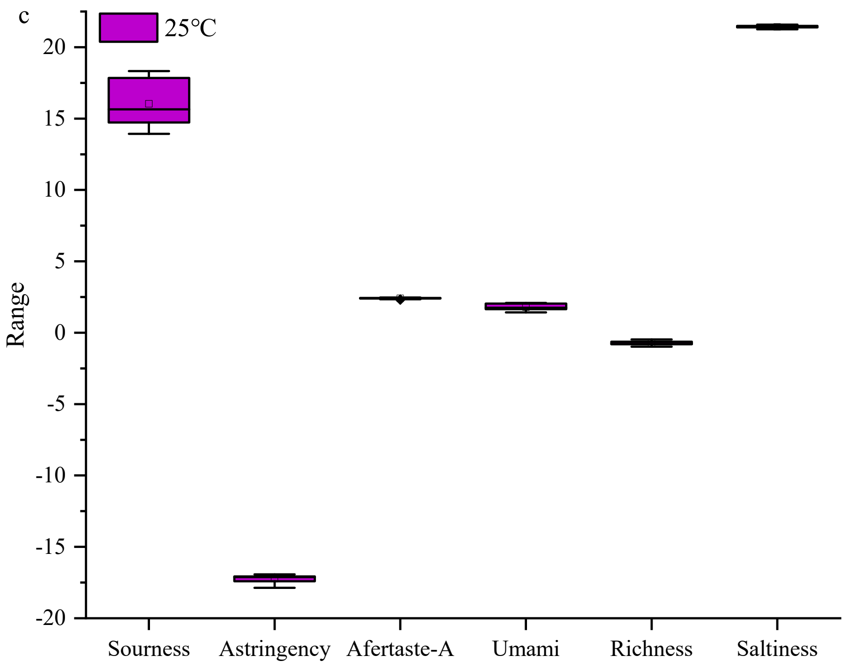

Figure S1. Taste changes of Sichuan sauerkraut under different storage conditions (a: storage condition is 45°C; b: storage condition is 35°C; c: storage condition is 25°C).
